# Topographic Cues Reveal Two Distinct Spreading Mechanisms in Blood Platelets

**DOI:** 10.1038/srep22357

**Published:** 2016-03-03

**Authors:** Rabea Sandmann, Sarah Köster

**Affiliations:** 1 Institute for X-Ray Physics, Georg-August-Universität Göttingen, Göttingen, 37077, Germany

## Abstract

Blood platelets are instrumental in blood clotting and are thus heavily involved in early wound closure. After adhering to a substrate they spread by forming protrusions like lamellipodia and filopodia. However, the interaction of these protrusions with the physical environment of platelets while spreading is not fully understood. Here we dynamically image platelets during this spreading process and compare their behavior on smooth and on structured substrates. In particular we analyze the temporal evolution of the spread area, the cell morphology and the dynamics of individual filopodia. Interestingly, the topographic cues enable us to distinguish two spreading mechanisms, one that is based on numerous persistent filopodia and one that rather involves lamellipodia. Filopodia-driven spreading coincides with a strong response of platelet morphology to the substrate topography during spreading, whereas lamellipodia-driven spreading does not. Thus, we quantify different degrees of filopodia formation in platelets and the influence of filopodia in spreading on structured substrates.

In mammals, blood loss in case of injuries is prevented by blood clotting. One key player in the blot clotting cascade are blood platelets, small cellular fragments, which circulate in the blood stream, adhere at the wounded site, spread and collectively form a blood clot that closes the wound temporarily. However, platelets do not only interact with other cells and tissues, but are thought to also be directly involved in the response of the body to implant materials^1^. In the healthy organism, blood clotting is precisely regulated, since both under- and overreaction are potentially life threatening.

During spreading, platelets extend different types of cellular protrusions, including filopodia and lamellipodia^2^. Filopodia are thin cellular protrusions containing bundles of actin filaments, whereas lamellipodia are laterally more extended and consist of a network of cross-linked actin filaments^3^. The different types of protrusions have different, equally important, physiological roles: filopodia connect individual platelets to each other^2^, whereas lamellipodia help the platelets to cover the wounded area^4^. In general, proteins that influence actin polymerization, like F-actin capping protein, Ena/VASP proteins and Diaphanous (a formin) are thought to determine whether cells form filopodia or lamellipodia^5^,^6^. Moreover, platelets can be triggered for example by thrombin^7^ and for murine platelets the activation of a thrombin receptor has been shown to be important for the regulation of filopodia formation^8^.

Another function that has been attributed to filopodia is the sensing of the extracellular environment^3^ and they may even be able to recognize the curvature of topographical features in the underlying substrate^9^. Moreover, Albuschies *et al*.^10^ showed that filopodia sense the topography of a substrate. This process is mediated by traction forces exterted by the filopodia which in turn influence the adhesive contacts and thus cell behavior and morphology. Thus, the role of filopodia and lamellipodia in spreading on micro-structured substrates has been investigated for mesenchymal stem cells where both filopodia and lamellipodia have been shown to react to the underlying topography and thus direct spreading^11^. For blood platelets, a role of filopodia in directing spreading on selectively coated substrates has been suggested and attributed to the sensory functions of filopodia^12^. Due to their great biomedical importance, the behavior of blood platelets on structured surfaces has mostly been studied with respect to implant design^1^,^13^,^14^,^15^,^16^,^17^,^18^,^19^. These studies were able to show an influence of substrate topography on the degree of platelet adhesion and activation. Despite these important studies of platelet spreading behavior and the discovery that platelets can spread with or without contribution of filopodia more than 30 years ago by Allen *et al*.^2^, it is to date not fully understood, how exactly cellular protrusions are involved in spreading of platelets on structured substrates.

Here, we employ a simplified topographic environment and dynamically investigate the spreading process by fluorescence microscopy. We analyze the temporal evolution of cell area, cell morphology and filopodia dynamics. Platelet morphology is strongly influenced by the topographic cues. Interestingly we find two spreading mechanisms, one that involves numerous filopodia and one that preferably shows lamellipodia. The topographic “disturbance” used in our experiments enables us to distinguish these mechanisms from each other. Thus, this study does not only shed light on the general role of protrusions in cell spreading but also suggests a role of filopodia in determining the morphology of blood platelets on structured substrates.

## Results

### Platelet area does not depend on substrate structure

In order to elucidate details of the involvement of cellular protrusions in blood platelet spreading, we perform time-lapse studies with a resolution of one frame per 1.5 s of membrane-stained platelets. We compare smooth and structured substrates. The topographic cues on the structured substrates are arranged in a square lattice of holes. The hole size is chosen small enough such that the platelets are unlikely to completely spread inside the holes. At the same time they are large enough to detect their influence on platelet morphology by visible light microscopy. All substrates are coated completely with fibrinogen to provide adhesion sites.

**Figure 1:**
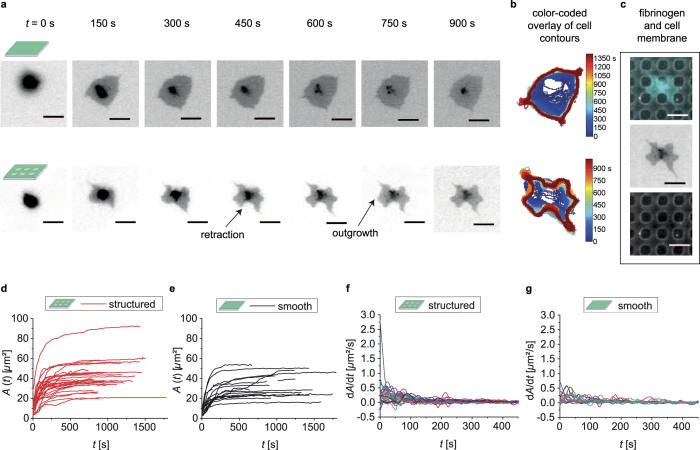
Spreading platelets on PDMS substrates. (**a**) Snapshots of the spreading process. Spreading on smooth substrates (top) occurs in a regular manner. On structured substrates (bottom), however, spreading is occasionally accompanied by retractions and outgrowth at the site of the holes (see arrows for examples). Shown is a cell that responds to the structured substrate by avoiding the holes at its periphery. Images show inverted gray scale fluorescence micrographs. (**b**) Color-coded overlays of the cell outlines (same cells as shown in (**a**)), from dark blue for early time-points and dark red for late time-points. (**c**) Fluorescence micrographs of the fibrinogen coating of the substrate and of the plasma membrane (same cell as in (a, bottom): overlay (top), membrane staining (center, inverted), substrate coating (bottom). (**d,e**) Moving average of spread area against time; cells spreading on structured ((**d**), 29 cells) and on smooth ((**e**), 16 cells) substrates show no difference in final spread area. (**f,g**) Derivative of area curves on structured and smooth substrates over time. Negative values below −0.05 *μ*m/s^2^, which may be caused by retractions, occur more often on structured substrates (165 times for 29 cells) than on smooth substrates (37 times for 16 cells). Micrographs for the cell on a structured substrate are rotated such that the main axes of the holes align with the main axes of the microscopy image (in ImageJ^34^ with bilinear interpolation). Scale bars indicate 5 *μ*m.

Figure 1a shows snap-shot images for different time-points for spreading on a smooth (top) and on a structured (bottom) substrate (see also the corresponding movies in the supplementary material; movie S1, movie S2). Figure 1b shows the cell outlines overlaid in a color-coded way from dark blue for early time-points to dark red for late time-points in the time-lapse series. In Fig. 1c the correlation between cell outline and underlying substrate structure is shown by fluorescence micrographs of the cell membrane (top: cyan, center: inverted grayscale) and the fibrinogen coating of the substrate (top and bottom: grayscale). Note that the overall brightness of the images of the cell membrane depends, e.g., on the time elapsed since staining or on unbound dye molecules in the cell suspension. However, contrast rather than absolute fluorescence intensity is key for our data analysis. The regions of higher fluorescence in the center of the cells, which appear as dark spots in these images, likely arise from heavily stained material stored in the cells. The increased amount of stained material may be due to granules inside the cell which are translocated to the cell center during spreading^4^, to remaining parts of the open canalicular system (OCS)^20^,^21^ or to a higher packing of the (wrinkled) membrane which smoothens during spreading^21^.

Differences in cell morphology are clearly visible even at early time points of spreading. Typically, spreading on smooth substrates appears regular, whereas on structured substrates spreading may lead to an irregular morphology with platelets avoiding the holes at their periphery. Furthermore, when already spread partially over a hole, cells on structured substrates may retract, as can be seen for example by comparing the images for 300 and 450 s or 750 and 900 s in Fig. 1a (bottom). The color-coded overlays in Fig. 1b illustrate a more dynamic spreading on structured substrates compared to spreading on smooth substrates.

To quantify our observations, we determine the temporal evolution of the cell area as a straightforward way to describe the spreading, shown in Fig. 1d,e. Interestingly, the final spread area is not influenced by the substrate topography for the substrates used here (see Fig. S1a in the supplementary material), in agreement with previous static data on fixed cells^22^. However, a closer look at the complete course of the temporal evolution of the spread area *A*(*t*) reveals retractions that are observed as “dips” (local minima) in the curves (see also Fig. S1b for examples in the supplementary material) and as negative values in the time derivative of the area as shown in Fig. 1f,g. These negative values in the derivatives occur more frequently on structured substrates than on smooth substrates indicating a larger influence of retractions.

### Two groups of platelets with different morphology can be distinguished on structured substrates

Although the area curves show little differences between spreading on structured and on smooth substrates, the morphologies may differ remarkably as can be seen in Fig. 1a,b. We thus analyze the spreading dynamics in an angle dependent manner (steps of 15°, see Fig. S2a in the supplementary material). We compare the values to results from an ellipse that has the same eccentricity, orientation and area as the cell, to capture effects of the outline roughness while disregarding influences of the non-circularity of the cell. When analyzing the eccentricity for the last time-point of each platelet on structured substrates, we find a mean value of 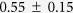 (s.d.; 29 platelets); on smooth substrates we find 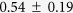 (s.d.; 16 platelets). A value of 0 corresponds to a circle and 1 to a line. Thus, on neither substrate the cells show a circular morphology on average.

**Figure 2:**
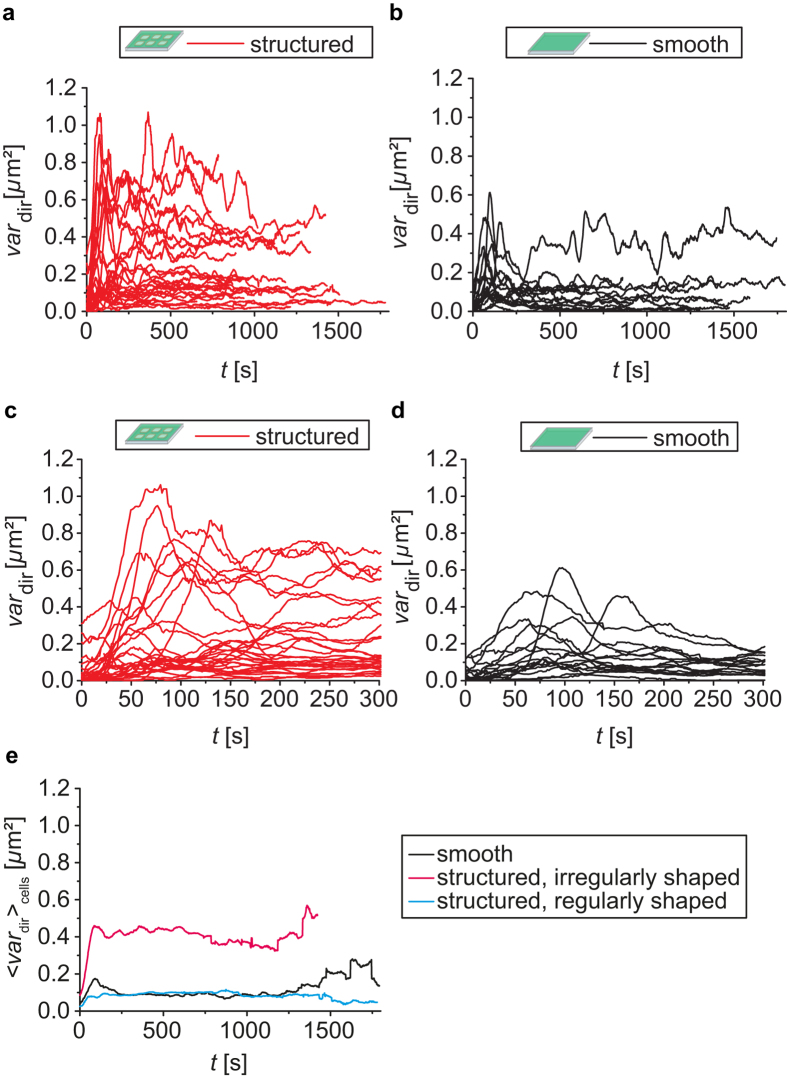
Angular (directional) variance of the spreading dynamics. Red: structured substrates, 29 cells; black: smooth substrates, 16 cells. (**a,b**) Plots of variance in angle 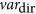 against time show a higher variance for several cells on structured substrates compared to the variance on smooth substrates. (**c,d**) Detailed plot of 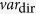 on structured and smooth substrates for early time points in spreading. (**e**) 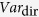 averaged over all cells 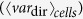 on smooth substrates (black), irregularly shaped platelets on structured substrates (pink) and regularly shaped platelets on structured substrates (light blue) showing that regularly shaped platelets on structured substrates show 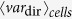 values similar to those of platelets on smooth substrates, while irregularly shaped platelets show higher values.

The vectors 
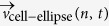
 between the ellipse and the cell outline with *n* being the angle and *t* the time point show local differences in spreading behavior. To quantify how much the cells deviate from the corresponding ellipse, we compute the variance between the moving average of the signed length 
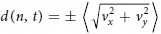
 of these vectors (positive sign when the cell outline lies on average outside the ellipse and negative when it lies on average inside, where 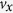 is the x-component of the vector and 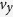 is the y-component) for each time point 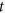:


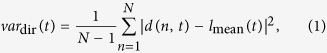


with the average of all moving-average treated signed lengths 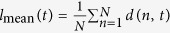 and the total number of angles *N *= 24. In Fig. 2a,b the values of 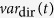 are plotted against time on structured (left, red) and on smooth substrates (right, black). On structured substrates several cells show high values for the variance, by contrast to only one single cell on the smooth substrates. This trend is visible from early time points of spreading on as shown in Fig. 2c,d and thus supports our observation described above for the cell morphology.

These differences in morphology throughout spreading together with the fixed final spread area, suggest that platelets have means to regulate their spreading area while adjusting their morphology to avoid the obstacles provided by the substrate. However, not all platelets on structured substrates show this behavior. Some platelets on structured substrates behave very similar to platelets on smooth substrate and show only moderate values for the variance during spreading. To further illustrate these two types of platelets on both smooth and on structured substrates, we refer to Fig. S3 in the supplementary material where the moving average of the signed lengths 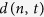 is shown. To describe our observations, we classify the platelets as *regularly shaped* if they show an average morphology throughout spreading that resembles the morphology of platelets on smooth substrates. As a threshold we define mean values of 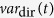 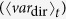 below 0.18 mm^2^. Platelets deviating stronger from an ellipse are classified as *irregularly shaped*. As can be seen in Fig. 2e regularly shaped platelets on structured substrates show very similar values for 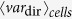 (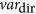 averaged over all cells in the category) as platelets on smooth substrates, while irregularly shaped platelets show higher values. When regarding the relative perimeter (perimeter_cell_/perimeter_ellipse_) in the last analyzed time-point of each time-lapse series, these two groups of platelets show different values as well: The regularly shaped platelets show relative perimeters that are similar to those of platelets on smooth substrates while the irregularly shaped platelets in general show higher values (see Fig. S4 in the supplementary material). Note, that although the morphology is clearly different for these two types of platelets on structured substrates, the groups cannot be separated by looking at the distribution of final area values (see Fig. S1c in the supplementary material).

### The two platelet groups show a different spreading behavior

**Figure 3:**
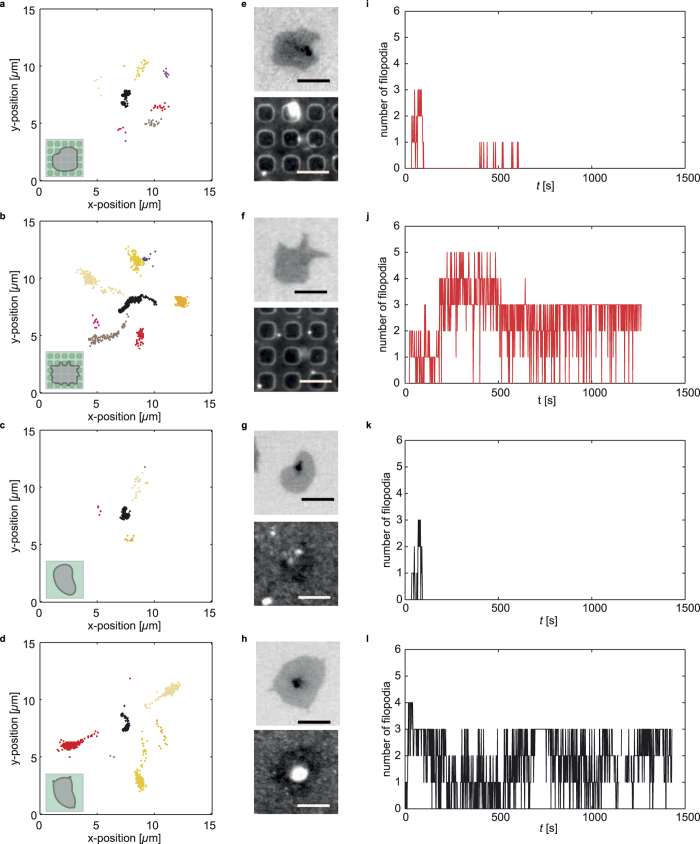
Filopodia behavior on different substrates. (**a–d**) Color-coded plots of sorted endpoints with the center of mass of the cell displayed as black crosses. (**e–h**) Micrographs of the cells (top) and the fibrinogen coating (bottom) after the time-lapse series is completed. Scale bars indicate 5 *μ*m. Micrographs for the cells on a structured substrate are rotated such that the main axes of the holes align with the main axes of the microscopy image (in ImageJ^34^ with bilinear interpolation). (**i–l**) Temporal evolution of filopodia endpoints. (**a,e,i**) Typical regularly shaped platelet on a structured substrate with quickly vanishing filopodia. (**b,f,j**) Typical irregularly shaped platelet on a structured substrate with numerous persistent filopodia. The micrographs reveal that the irregularly shaped platelet shows a morphology that is very different from those on smooth substrates. (**c,g,k**) Typical platelet on a smooth substrate showing filopodia, which do not persist for a long time. (**d,h,l**) Typical platelet on a smooth substrate showing persistent filopodia. Although the dynamic behavior of the filopodia is different for theses two examples of platelets on smooth substrates, the final morphology is similar as can be seen in the micrographs.

The question remains, why some platelets show a regular and some show an irregular shape on structured substrates. Earlier studies hint at an involvement of cellular protrusions in sensing of and response to the environment^3^,^9^,^12^. Thus, we further investigate filopodia dynamics by directly tracking them and plotting their tips in color coded groups, as shown in Fig. 3. In this representation of the filopodia, differences between individual platelets on structured substrates are clearly visible (Fig. 3a,b). While in Fig. 3a, the colored groups contain fewer endpoints and are in general less extended, in Fig. 3b, they are elongated and directed radially from the cell center. Interestingly, we observe the same two types of platelets also on smooth substrates (Fig. 3c,d). This phenomenon becomes even clearer when plotting the number of filopodia against time. Platelets either protrude filopodia that vanish after a short time (Fig. 3i,k) or show more persistent filopodia (Fig. 3j,l). However, the micrographs after the time-lapse series is completed (inverted gray scale micrographs Fig. 3g,h) show a similar morphology for the cells on smooth substrates.

In summary, these observations suggest that the dynamics of filopodia differ more between two distinct groups of platelets than between platelets on substrates with or without topographic disturbances. Therefore, we hypothesize that two spreading mechanisms exist for platelets. On smooth substrates, from merely analyzing the cell morphology the results from both mechanisms look very similar; however, by dynamically tracking individual filopodia, they can be distinguished. By contrast, on structured substrates, which impose a topographic cue on the spreading platelets, increased numbers of dynamic filopodia coincide with a stronger response to the structure.

To test this hypothesis we plot the mean number of filopodia per video frame as well as the standard deviation against time. When comparing the mean number of filopodia of all platelets analyzed on smooth (4a, gray) and all platelets analyzed on structured substrates (4b, black), no clear difference is observed. In the beginning, the mean number rapidly increases to about 2 and then slowly decreases to about 1. The similar mean numbers of filopodia show that the distribution of filopodia numbers is the same for the whole ensemble of platelets which we studied here. We analyzed movies of 45 platelets in total, including 29 on structured substrates (irregularly shaped: 13 platelets, regularly shaped: 16 platelets).

**Figure 4:**
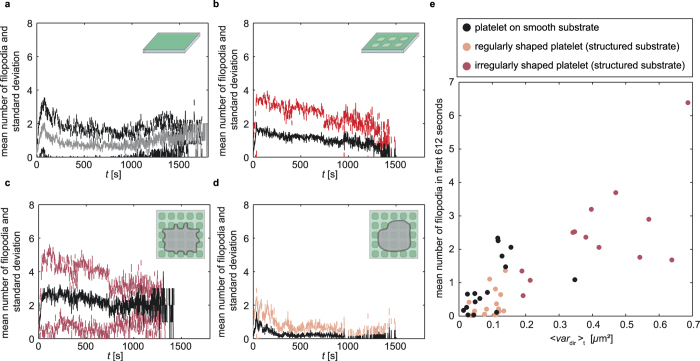
Temporal evolution of mean filopodia numbers. (**a**) The mean numbers of filopodia for all platelets on smooth (16 cells, mean: gray, standard deviation: black) and (**b**) on structured substrates (29 cells, mean: black, standard deviation: red) do not differ strongly. (**c,d**) If the platelets on structured substrates are subdivided into (**c**) irregularly shaped (13 out of 29 cells, mean: black, standard deviation: pink) and (**d**) regularly shaped platelets (16 out of 29 cells, , mean: black, standard deviation: light orange), clear differences are observed. (**e**) Mean number of filopodia (averaged over the first 612 video frames) plotted against the mean of the angular variance 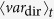.

For the structured substrates, we can assign the platelets to either those that have a regular or those that have an irregular morphology, as shown in Fig. 4c,d. Here, we find clear differences for the mean numbers as well as the standard deviations. Irregularly shaped platelets (4c, black) show the highest mean number of filopodia of all examined groups while regularly shaped platelets (4d, black) show the lowest mean number of filopodia. Interestingly, filopodia vanish nearly completely in the case of regularly shaped platelets after about four minutes, whereas for irregularly shaped platelets filopodia persist throughout the whole time-lapse series. The clear differences in mean number support the idea that two spreading mechanisms for platelets exist, where the number of filopodia is correlated with the degree of response on structured substrates. On smooth substrates the different types of platelets cannot be distinguished via morphology as in both cases the morphology is quite similar (see also Fig. 3g,h). Thus, there is no obvious parameter except for the number of filopodia themselves to distinguish between different types of platelets. In all groups plotted in Fig. 4, a fast initial increase in mean number of filopodia is observed. Thus, filopodia formation itself is initiated very fast and likely independently of the substrate type.

In Fig. 4e we compare the mean variance of spreading in different angles over the whole time-lapse series 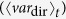 with the number of filopodia averaged over the first 612 s. For irregularly shaped platelets (pink) the mean number of filopodia increases with increasing 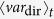 and reaches high values, whereas for regularly shaped platelets (light orange) both the mean number of filopodia and 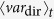 remain low similar to the values for the smooth substrate.

### Possible spreading scenarios on structured substrates

**Figure 5:**
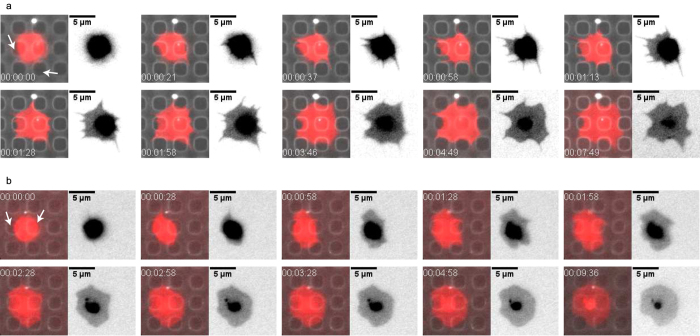
Spreading behaviors on structured substrates. Micrographs are rotated such that the main axes of the holes align with the main axes of the microscopy image (in ImageJ^34^ with bilinear interpolation). Fluorescence micrographs show overlays of cell membrane staining (red) and fibrinogen coating (gray) of the substrate (left hand side); additionally inverted gray scale images of the cell membrane (right hand side) are shown. The time-point in the spreading process is shown in hh:mm:ss format. (**a**) The platelet shown here has been classified as irregularly shaped and shows several filopodia as well as retractions over the holes during spreading. (**b**) This platelet has been classified as regularly shaped and shows only very few filopodia while mainly spreading via lamellipodia.

One possible explanation why differences in filopodia formation and morphology coincide on structured substrates but not on smooth substrates could be that spreading via filopodia is disturbed on structured substrates due to the topographic cues. Indeed, for the platelet shown in 5a (see also movie S3 in the supplementary material) showing numerous filopodia during spreading, retractions over the holes can be observed (see positions marked by white arrows in the first image). First, the platelet extends filopodia along the interspaces or over the holes; subsequently, lamellipodia form in between the filopodia and cover the holes partly; finally, the lamellipodia retract over the holes. In Fig. 5b (see also movie S4 in the supplementary material) a different situation is depicted. Here, the platelet spreads mainly by extending lamellipodia. As can be seen for the positions marked by white arrows in the first picture, these lamellipodia extend over the holes and finally cover the complete hole, so that the cell does not show a strong response to the substrate.

## Discussion

Dynamic imaging of spreading platelets at adapted temporal and spatial resolution while employing structured substrates as a tool, reveals two mechanisms for spreading of platelets which differ in their involvement of persistent filopodia. Among these two mechanisms, the one that is characterized by the involvement of numerous, persistent filopodia leads to much stronger responses to the substrate structure, while the final spread area remains the same for both mechanisms. On smooth substrates the average morphology is similar for all cases and the two mechanisms can only be distinguished by tracking filopodia dynamics throughout the time-lapse series.

**Figure 6:**
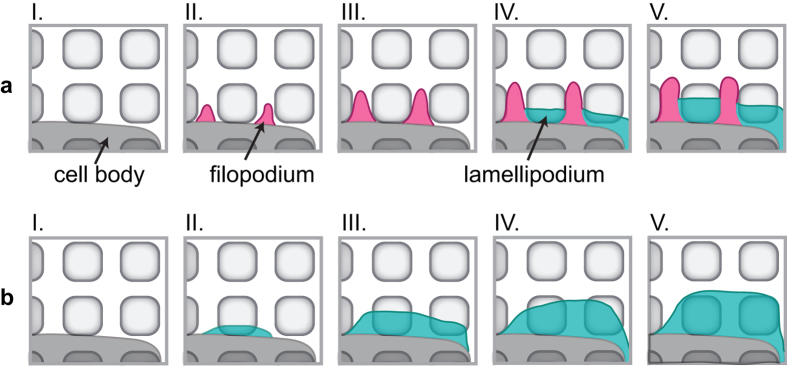
Sketch of possible spreading scenarios. (**a**) Spreading involving filopodia (magenta). First, filopodia form between the holes (I.–III.). Then, spreading via lamellipodia (cyan) sets in (IV.). A lamellipodium is dragged forward in between the filopodia to cover the hole but is not able to cover the hole completely and thus this part of the cell remains unstable and leads to a morphology that is very different from the one found on smooth substrates. (**b**) Spreading mainly achieved via lamellipodia. A lamellipodium (cyan) covers the holes completely during spreading (I.–V.) and the cell is more stable at this position as more binding sites can be established early on. Thus, this type of spreading may lead to a morphology that is similar to that found on smooth substrates.

By comparing the “irregularity” of the cell morphology (quantified by 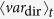) and the number of filopodia we find a correlation factor of 0.85 between 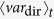 (averaged over the whole time-lapse series) and the mean number of filopodia in the first 612 seconds. For platelets on smooth substrates this correlation factor is only 0.49. Here, the correlation is calculated as 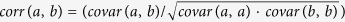 with the mean number of filopodia in the first 612 seconds *a*, the mean of 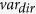 over the whole time-lapse series *b* and the covariance matrix *covar*. These data along with direct imaging of the different ways of spreading as shown in Fig. 5 lead us to suggest the following spreading process via filopodia or lamellipodia, respectively (illustrated in Fig. 6): If spreading is achieved with the contribution of filopodia, these filopodia are likely positioned between the holes in the substrate or reach over a hole to connect to the next interspace where they can form contact points to the substrates (Fig. 6a, I.–III.). In a subsequent step, lamellipodia may be extended in between the filopodia to cover the holes (Fig. 6a, IV.–V.). However, complete coverage of the hole may or may not be achieved by this process. Instabilities and retractions at the positions of the holes may occur and finally leave the platelet in a spread state in which the cell avoids the holes at its periphery. Cells displaying this type of morphology differ strongly in their morphology from platelets on smooth substrates. If, by contrast, the platelet spreads mostly without filopodia, it extends mainly lamellipodia early on (Fig. 6b, II. and Fig. 5b) and may thus establish many binding sites already in the beginning of spreading. The lamellipodia may cover the holes and establish adhesions at the opposite side of the hole. This process will render the platelet stable at the position of the hole and thus lead to a morphology similar to that on smooth substrates.

A similar coincidence between different spreading modes and filopodia formation has been observed for fibroblasts, albeit on smooth glass surfaces^23^,^24^. These fibroblasts were part of the same cell culture but could be divided into anisotropically spreading cells with filopodia and isotropically spreading cells without filopodia. During anisotropic spreading the fibroblasts showed membrane ruffles and edge retractions. Thus, the irregularly shaped platelets discussed here might follow a similar spreading mechanism as the anisotropically spreading fibroblasts described in these publications.

It does remain an open question, however, what triggers filopodia development or suppression in platelets. One possibility could be the activation of thrombin receptors as has been described for murine platelets^8^. Mouse platelets with activated thrombin receptors (PAR4) showed fewer filopodia than platelets without activated receptors. Furthermore the authors report that the temporal evolution of filopodia numbers was affected and receptor activation led to disappearance of filopodia after four minutes. This time scale is strikingly similar to what we find for our system, where for regularly shaped platelets on structured substrates, the filopodia vanish after four minutes as well. However, both regularly and irregularly shaped platelets can be observed within the same sample preparation in our experiments and thus these differences in thrombin stimulation can only arise from either differences in thrombin receptor density on the specific platelet or from different thrombin concentrations the individual platelets experience. In this context also the residence time in the solution before the platelets attach to the substrate may play a role and regulate the amount of thrombin the platelet experiences.

Another explanation for the different filopodia numbers may be found in variation in copy numbers of certain proteins in these tiny cells. Indeed, it has been shown for example for murine fibroblasts that differences in protein content of F-actin capping protein and Ena/VASP proteins determine whether filopodia or lamellipodia form^5^ while Diaphanous and Ena seem to regulate this process in D16C3 cells^6^. Platelets are very small cells and show a rather large variation in cell diameter (diameter of quiescent platelets: about 2–5 *μ*m^25^) and thus comparably small variation in protein number may lead to large relative differences. Furthermore, platelets have been shown to produce proteins during storage^26^ and differences in protein levels may arise from variations in protein production during aging of the platelet in the blood stream.

From a physiological point of view, it does indeed make sense that two types of platelets exist, one that expresses more filopodia and one that shows rather few. Spreading mostly via lamellipodia likely enables the platelet to cover the wounded site even if it does not adhere to a completely smooth and even surface. Such uneven surfaces are found everywhere in the body and are most likely pronounced at wounded site where the cell layers are disrupted. By contrast those platelets that exhibit more filopodia can recruit other platelets to the wounded site^4^ and furthermore may stabilize the blood clot by intertwining protrusions^2^.

## Conclusion

To summarize and conclude, our results obtained from human blood platelets spreading on structured and on smooth substrates reveal the existence of two spreading mechanism of platelets with or without the involvement of numerous persistent filopodia, respectively. The use of topographically structured substrates in combination with time-resolved imaging enables us to show that the filopodia-rich platelet group coincides with irregular morphology and strong response to the substrate structure. By contrast, when observing the spreading process on smooth substrates or when looking only at the end-state of the spreading, the correlation between filopodia formation and response to the substrate remains hidden.

Filopodia formation may be important for the physiological role of each of the two groups we found here since lamellipodia may help the platelets to cover the wound more efficiently, whereas platelets forming filopodia may primarily recruit other platelets. Future experiments involving defined spatio-temporal application of thrombin could shed light on a possible involvement of the thrombin receptors in filopodia formation as it has already been investigated for murine platelets. Such an experiment could be performed by employing photo-activatable thrombin^27^,^28^,^29^ in combination with microfluidic methods. A better understanding of the dynamics of the platelet spreading and adherence process does not only lead to novel insights into early steps of wound healing but also brings forward the functional design of implants which are incorporated in the body by a platelet “scaffold” for other cell types to adhere to.

## Methods

### Fabrication of Substrates

A detailed description of the substrate fabrication was published elsewhere^22^. In brief, PDMS replicates (thickness 45 (±5) *μ*m (s.d.) were produced from a silicon wafer structured by photolithography^30^,^31^ to an approximate height of 540 (±10) nm (s.d.). The PDMS substrates contained holes with 2.8 (±0.1) *μ*m diameter (s.d.), 1.1 (±0.1) *μ*m (s.d.) apart. After fibrinogen-coating (fibrinogen from human plasma, Alexa Fluor 488 conjugate, excitation ~495 nm, emission: ~519 nm, Invitrogen, Darmstadt, Germany) of the complete surface, the substrates were stored in PBS until used.

### Platelet Isolation and Plasma Membrane Staining

A detailed description of platelet isolation was published elsewhere^32^. The experiments were conducted with the consent of the Ethics Committee of the University of Göttingen and its ethical vote 11/11/09. The centrifugation steps were performed at 20–21 °C and the cell pellet was dissolved in Hepes-Tyrode buffer in the final step of purification (134 mM NaCl, 12 mM NaHCO_3_, 2.9 mM KCl, 1 mM MgCl_2_, 5 mM HEPES, 5 mM Glucose, 0.34 mM NaH_2_PO_4_, pH 7.4, supplemented with 5 mg ml^−1 ^BSA (Macs BSA stock solution, Milteny Biotech, Bergisch Gladbach, Germany)). CellMask DeepRed dye (excitation: ~649 nm, emission: ~666 nm, Life Technologies GmbH, Darmstadt, Germany) was used to stain the plasma membrane of the platelets. Since platelets are anucleate, it is not straightforward to transfect them for fluorescent actin and focal adhesion proteins. A “staining solution” was prepared from Hepes-Tyrode buffer (supplemented with 5 mg ml^−1 ^BSA) and CellMask DeepRed dye to obtain a final dye concentration of 2.5 *μ*g ml^−1^. Approximately 995 *μ*l of staining solution were mixed with about 5 *μ*l of cell suspension (final platelet concentration 2 × 10^7 ^cells ml^−1^). The mixture was incubated for 6 minutes at 37 °C and 5% CO_2_. Prostaglandin E_1_ solution (Cayman Chemical Company, Ann Harbor, Michigan, USA) at a final concentration of about 2.7 *μ*g ml^−1^ was added and the solution was centrifuged for 5 minutes at 480 × g (20–21 °C). Finally, the cell pellet was resuspended in Hepes-Tyrode buffer with 5 mg ml^−1^ BSA. The experiments were performed on the basis of three different platelet isolations (2 for the structured substrates and 3 for the smooth substrates). 5 different substrates have been used for the experiments on structured substrates and 4 different substrates have been used for the experiments on smooth substrates.

### Image Acquisition

The substrates used for live imaging of membrane-stained platelets were washed three times with Hepes-Tyrode buffer with 5 mg ml^−1^ BSA before usage. In order to observe platelet spreading in close-to-physiological-conditions, experiments were performed using an on stage incubation chamber (37 °C, 5% CO_2_, model INUG2E-ONICS, Incubation System for Microscopes, Tokai Hit, Ltd., Gendoji-cho, Fujinomiya-shi, Shizuoka-ken, Japan). 150 *μ*l stained platelet solution was added to the substrate. To trigger spreading, 50 *μ*l thrombin solution (16 u ml^−1^; thrombin from human plasma, Sigma-Aldrich, St. Louis, Missouri, USA) were added. Imaging was performed using an inverted research microscope (IX81, Olympus, Hamburg, Germany) equipped with a 60× silicone-oil immersion objective ULSAPO60XS2 (Olympus), a Cy5/FITC dualband filter (AHF Analysentechnik AG, Tübingen, Germany) and an ORCA-R2 CCD-camera (Hamamatsu Photonics Deutschland GmbH, Herrsching am Ammersee, Germany). To be able to correlate platelet spreading to the substrate structure, fluorescence images of both cell membrane and substrate coating were recorded every 1.5 s.

### Data Analysis

If not indicated otherwise, data analysis was performed using MATLAB programs (MATLAB R2009b, TheMathworks, Natick, MA, USA). Details of the data analysis can be found in the supplementary information. In brief, the fluorescence micrographs were denoised and the cell outline was detected by the Canny edge detection method^33^. These outlines were compared to the original fluorescence images in ImageJ^34^ to manually add non-detected regions. Thereafter, the binarized images were corrected for drift by cross-correlation of images of the substrate coating. The images were rotated to align the axes of the holes in the substrates with the image axes.

In order to determine platelet morphology, the points of intersection, 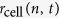, between the cell outline and 12 straight lines through the mean center of mass of the cell, *i.e*. for 24 angles (*n *∈ [0°, 365°]), as well as the points of intersection, 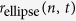, between an ellipse having the same area, orientation and eccentricity as the cell and the same straight lines were determined. Subsequently, the lengths of the vectors between the points of intersection of 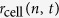 and 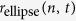 as well as the *x*− and *y*−values of these vectors were determined and denoised by computing the moving average including all values in the interval 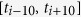. Finally, the directional variance 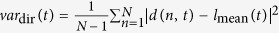 with 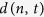 being the moving average treated signed length of the vectors between cell and ellipse, 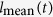 the average of all moving average treated signed lengths, *n* the individual angle and *N* = 24 the total number of angles was calculated for each time-point *t*. If the mean of 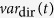 over all analyzed time-points 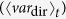 was larger than 0.18 *μ*m^2^, the platelet was classified as irregularly shaped to distinguish it from regularly shaped platelets that spread with similar average morphology as platelets on smooth substrates.

The endpoints of filopodia were detected by a combination of length and curvature constraints. For the length constraints, the distance from center of mass to the cell outline was computed and compared to points left and right of the considered point along the outline. The curvature values along the outline were computed from a spline-approximated cell outline. If both curvature and length constraints were met, the position was registered as a possible endpoint. If detected endpoints lay too close together only the one with the largest distance from the center of mass was kept. The endpoints were grouped by considering the distances between the endpoints and finally merging groups that lay too close together. The mean numbers and standard deviations of the number of filopodia for all analyzed platelets on smooth (16 cells) and all analyzed cells on structured substrates (29 cells) as well as for the sub-groups of regularly shaped cells on structured substrates (16 cells) and irregularly shaped cells on structured substrates (13 cells) were determined.

## Additional Information

**How to cite this article**: Sandmann, R. and Köster, S. Topographic Cues Reveal Two Distinct Spreading Mechanisms in Blood Platelets. *Sci. Rep*. **6**, 22357; doi: 10.1038/srep22357 (2016).

## Supplementary Material

Supplementary Information

Supplementary Movie S1

Supplementary Movie S2

Supplementary Movie S3

Supplementary Movie S4
