# Supplementary material for: Topographic Cues Reveal Two Distinct Spreading Mechanisms in Blood Platelets
Source: Sci Rep. 2016 Mar 3;6:22357. doi: 10.1038/srep22357 (PMC4776100; doi:10.1038/srep22357)
Supplement: Supplementary Information [file srep22357-s1.pdf]

Topographic Cues Reveal Two Distinct Spreading Mechanisms in Blood Platelets - Supplementary Information

Rabea Sandmann[†] and Sarah Köster[†*]

Additional Data

In figure S1a the mean final values for the cell area (calculated from the last 50 points of the moving-average-treated data for the cell area) are shown as a box-plot. As is visible from the outermost points of the whiskers, the range of final area values is similar for both cases (structured and smooth substrates). In figure S1b area curves in which retractions are visible by area dips are shown. The starting points of these dips are marked by black arrows. These dips do not persist for longer than a few minutes and are then compensated for. As shown in figure S1c, the area curves for irregularly shaped platelets (pink) and for regularly shaped platelets (cyan) show no large differences in final area.

In figure S3 the color-coded signed lengths $d(n,t)$ of the vectors (moving average treated) between cell and ellipse are shown for different time-points t (y-axis) and different angles n (x-axis) for cells on structured substrates (a,b) and cells on smooth substrates (c,d). Two behaviors of platelets on each substrate type are displayed. In figure S3a an irregularly shaped cell with alternating protrusions and invaginations is shown, while figure S3b shows a regularly shaped cell. Although the cell in figure S3c also shows alternating protrusions and invaginations, these differences vanish over time while in S3a they become amplified.

In figure S4 the relative perimeter in the last analyzed time-point of the time-lapse series is plotted for the different spreading mechanisms (on smooth substrates (black), regularly shaped platelets on structured substrates (light orange) and irregularly shaped platelets on structured substrates (pink)). The relative perimeters for regularly shaped platelets resemble those for platelets on smooth substrates while the relative perimeters for irregularly shaped platelets are in general higher.

Analysis

If not indicated otherwise, data analysis was performed using MATLAB programs (MATLAB R2009b, TheMathworks, Natick, MA, USA). Cells were not analyzed in the following cases: cells positioned on a structured part of the substrate but too close to the smooth part and *vice versa*; cells still showing a large, bright, central spot in the membrane-staining after the time-lapse series had ended, since these cells likely did not spread completely; cells showing a clear and long-lasting contact to another cell; cells that could be analyzed for less than 400 images, *i.e.* 600 seconds.

Image binarization and drift correction

In order to analyze cell spreading, the outline for each time point was extracted from the micrographs. The images were filtered by the *wiener2* function in a neighborhood of 7 × 7 pixels to reduce noise. The *canny* algorithm[1] (option of *edge* function) with a low threshhold of 0.02 and a high threshold of 0.07 was employed to detect simultaneously weak and strong edges in the image. Overlays of the detected edges with the original fluorescence images in ImageJ[4] revealed undetected regions which were then

added manually. Outlines were filled to create a mask of the cell.
The drift between images of a time-lapse series was corrected for by computing the cross-correlation between each image in the series and a reference image as sketched in Ref.[5]. The drift was calculated based on the images of the substrate coating, which did not change as much as the cell images, and then corrected for in the cell images by linear translocation as described in Ref.[2].

Analysis of spreading along different angles

Local analysis of spreading was performed by computing the spreading along different angles and normalized by computing the difference to an ellipse with the same area, orientation and eccentricity as the cell. The binarized image of the cell was rotated by the *imrotate* function (bilinear interpolation) to align the rows of holes in the substrate with the image borders and thus ensure comparability for cells on different substrates. The images were binarized again as due to the interpolation in the rotation of the binarized image a grayscale image arises. The cell area and center of mass as well as the major and minor axes of an ellipse that has the same normalized second central moments as the cell were calculated using the functions *bwlabel* and *regionprops*. To obtain an ellipse that has the same area as the cell, the major and minor axis were rescaled while keeping the eccentricity constant, since the ellipse calculated by Matlab does not automatically have the same area as the cell. 120 points lying on the ellipse were calculated as described in Ref.[3].
Straight lines enclosing different angles with the horizontal axis (0° to 165° in increments of 15°) and passing through the mean center of mass of the cell (averaged over the whole movie) were computed. The outline of the cell was extracted *via* the *bwperim* function (8-connected neighborhood). The points of intersection, $r_{\mathrm{cell}}(n,t)$ between the cell outline and the straight lines were computed for each time point t and each angle n. Images were only analyzed if all points on a cell outline were situated at least 2 pixel away from the mean center of mass and the mean center of mass lay inside the cell, as otherwise the results would be largely influenced by the movement of the cell.
Points lying on the straight lines are denoted as s_{line} and points lying on the cell outline are denoted as s_{cell} in the following. Points of intersection $r_{\mathrm{cell}}(n,t)$ between the cell outline and the straight lines were detected by comparing points on the lines s_{line} and on the cell outline s_{cell}: If a point s_{cell} was found, whose coordinates were the same as the rounded coordinates of a point on the line s_{line}, a point $r_{\mathrm{cell}}(n,t)$ was registered (figure S2a, red points). For each straight line and each time point two points of intersection - one on each site of the cell or ellipse - were searched.
To normalize the spreading in different directions and account for contributions of cell shape, points of intersection with the straight lines on the ellipse, $r_{\mathrm{ellipse}}(n,t)$, were detected. Here, if points s_{line} and $s_{ellipse}$ with a distance smaller than 1.5 pixels were found, a point $r_{\mathrm{ellipse}}(n,t)$ was registered. A plot of the points detected along different directions on the ellipse is shown in figure S2a (cyan squares).
The vertical direction corresponding to 90° or 270°, respectively, is an exception in the analysis as in this case two points on the cell outline were searched whose x-value cor-

responded to the rounded x-value of the mean center of mass. For the ellipse, points of intersection were registered if a point $s_{ellipse}$ did not differ more than 1 pixel from the x-value of the mean center of mass.
The vectors $\vec{v}_{cell-ellipse}(n,t)$ between points $r_{\text{cell}}(n,t)$ and $r_{\text{ellipse}}(n,t)$ were calculated (figure S2a), magenta vectors). Moving averages of cell area $A(t)$, the x- and y- values and the lengths of the vectors $\vec{v}_{cell-ellipse}(n,t)$ were calculated for each frame t_i by averaging the points in the interval $[t_{i-10}, t_{i+10}]$. A negative norm of the vector $\vec{v}_{cell-ellipse}(n,t)$ corresponds to an invagination - the cell lies inside of the ellipse; a positive norm corresponds to a protrusion - the cell lies outside of the ellipse. $d(n,t)$ denotes the moving average of the signed lengths of the vector $\vec{v}_{cell-ellipse}(n,t)$ at time point t and direction n. The variance between the moving average of the signed lengths $d(n, t = const.)$ of different vectors $\vec{v}_{cell-ellipse}(n,t)$ at one time point, $var_{dir}(t)$, was calculated as sketched in figure S2b.

Calculation of dA/dt

The original data (without moving-average-treatment) for the cell areas were interpolated in OriginPro 8.5G (OriginLab, Northampton, MA, USA) with the interpolate option (method = linear) and afterwards differentiated (differentiation order = 1, smoothing: polynomial order = 2 over 21 points).

Calculation of the relative perimeter

For the relative perimeter, the perimeter of the binarized cell in the last time-point of the time-lapse series as well as the major ($2a$) and minor axis ($2b$) of the corresponding ellipse were calculated in ImageJ[4]. The perimeter of the ellipse was approximated based on the semi-major and semi-minor axis of the ellipse (a and b, respectively) via following equation[6]:

$$\text{perimeter}_{\text{ellipse}} = \pi * (a+b)\left(1 + \frac{3*\lambda^2}{10+\sqrt{4-3*\lambda^2}}\right)$$

with $\lambda = (a-b)/(a+b)$.
The relative perimeter was computed as $\text{perimeter}_{\text{cell}}/\text{perimeter}_{\text{ellipse}}$ and plotted in OriginPro 8.5G (OriginLab) via the box-plot option.

Detailed description of detection of filopodia endpoints

In order to detect the endpoints of filopodia, the binarized images of the cells were rotated with bilinear interpolation in order to align the axes of holes with the image axes. The membrane curvature was calculated from the spline-fitted points on the cell outline. Furthermore, the distances from the center of mass of the cell to the points lying on the cell outline, $l(r_1)$, were determined. We defined two constraints for the filopodia, a length constraint and a curvature constraint, as shown in figure S5d.
For the length constraint, each point on the cell outline r_1 was compared to a point

lying 11 points left along the outline r_{left} and a point lying 11 points right along the outline r_{right} with periodic boundary conditions. In order to be considered as an endpoint, $l(r_1)$ had to be larger than $(l(r_{\text{left}})+l(r_{\text{right}}))/2+5$ pixels and simultaneously larger than $l(r_{\text{left}})$ and than $l(r_{\text{right}})$. If the cell was nearly completely spread and nearly circular, *i.e.* the minimal distance of all points on the cell outline l_{min} was equal or larger than $0.9 * (A_{\text{fin}}/\pi)^{0.5}$ with A_{fin} being the final cell area, the length $l(r_1)$ had to be larger than twice the mean length l_{mean}. Otherwise, *i.e.* if the cell was not close to reaching its final area and not nearly circular, no further length constraint was applied.

For points on the cell outline that fulfilled these length constraints, the curvature constraint was tested: If the mean curvature c_{mean} of the whole cell outline was larger than $0.55\,\mu\text{m}^{-1}$ and the maximum curvature was larger than $2.5\,\mu\text{m}^{-1}$, the curvature for the possible endpoint s had to be equal or larger than $0.2 * c_{\text{max}}$. If c_{mean} and c_{max} were smaller than the values defined above, the curvature had to be equal or larger than $0.9 * c_{\text{max}}$. If all lengths and curvature conditions were fulfilled and the possible endpoint lay at least 10 pixels apart from any other detected endpoint, this point was registered as an endpoint of a filopodium. If other detected endpoints lay in closer vicinity, only the point with the largest length $l(r_1)$ was considered as an endpoint.

In order to group the detected endpoints of filopodia from all images in the time series, the positions of new endpoints were compared to the positions of endpoints in the former images (see figure S6). If at least one endpoint of a group from a former image was situated as close as 8 pixels to the new endpoint and no endpoint had already been assigned to the group for this time-point, the new endpoint was assigned to the group. Furthermore, each endpoint was assigned to the closest possible group of endpoints. If no groups were found that matched the conditions, a new groups was opened and the endpoint was assigned to this group. When all endpoints had been assigned, it was tested if groups of endpoints should be merged since they actually might represent the same filopodium (see figure S6b). To this end, groups of endpoints for which the x- and y-values of the mean centers of mass of these groups were situated less than 20 pixels apart were considered for merging. If the distance between any point of one group and any point of the other groups was less than 5 pixels and at no time-point two different endpoints would be assigned to the group by merging it with the other groups, these groups were merged.

The mean numbers and standard deviations of the number of filopodia for all analyzed platelets on smooth (16 cells) and all analyzed cells on structured substrates (29 cells) as well as for the sub-groups of regularly shaped cells on structured substrates (16 cells) and irregularly shaped cells on structured substrates (13 cells) were determined.

Calculation of the mean eccentricity and the correlation factor between the mean of $var_{dir}(t)$ and the number of filopodia in the first 612 seconds

The eccentricity for the last time-point of each image series was calculated through the *regionsprops* (option *eccentricity*) function from the binarized, unrotated images. The mean and the standard deviation for these values were calculated for platelets on structured and platelets on smooth substrates individually.

The correlation factor between the mean of $var_{dir}(t)$ ($< var_{dir} >_t$) and the number of filopodia was calculated by the *corrcoef* function.

Bibliography

[1] J. Canny. A computational approach to edge detection. *IEEE Transactions on Pattern Analysis and Machine Intelligence*, 8(6):679–698, 1986. doi: 10.1109/TPAMI.1986.4767851.

[2] S. Eddins. Spatial transformations: Translation confusion: Matlab central, Steve on image processing, 2006. URL `http://blogs.mathworks.com/steve/2006/07/07/spatial-transformations-translation-confusion/?s_tid=Blog_Steve_Category`.

[3] S. Eddins. Visualizing regionprops ellipse measurements: Matlab central, Steve on image processing, 2010. URL `http://blogs.mathworks.com/steve/2010/07/30/visualizing-regionprops-ellipse-measurements/`.

[4] C. A. Schneider, W. S. Rasband, and K. W. Eliceiri. NIH Image to ImageJ: 25 years of image analysis. *Nature Methods*, 9(7):671–675, 2012.

[5] The MathWorks, Inc. Registering an image using normalized cross-correlation. URL `http://www.mathworks.de/products/demos/image/cross_correlation/imreg.html`.

[6] M. B. Villarino. A note on the accuracy of Ramanujan's approximative formula for the perimter of an ellipse. *Journal of Inequalities in Pure and Applied Mathematics*, 7(1):article 21, 2006.

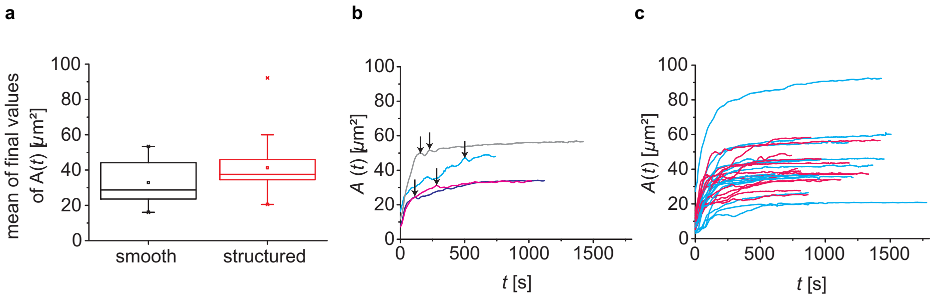

Figure S1: Temporal evolution of the spreading area. (a) The mean final area for each analyzed cell was calculated as mean of the last 50 time-points of the moving average treated data and plotted in OriginPro 8.5G (OriginLab, Northampton, MA, USA) via the box-plot option. The mean values are shown as squares while the lower and upper borders of the box show the values for which 25% or 75%, respectively, of all values lie under this value. The whisker length is determined by the smallest (lower whisker) and largest value (upper whisker) that lies in the range [lower border of box - (1.5 · size of box), upper border of box + (1.5 · size of box)]. The range of values is similar for cells on structured and on smooth substrates. (b) Curves of the moving average of $A(t)$ on structured substrates in which retractions are visible. Starting points of area dips are marked by arrows. The area loss due to the retractions is compensated for in a few minutes. (c) The distribution of final areas are similar for regularly (cyan) and irregularly (pink) shaped platelets on structured substrates.

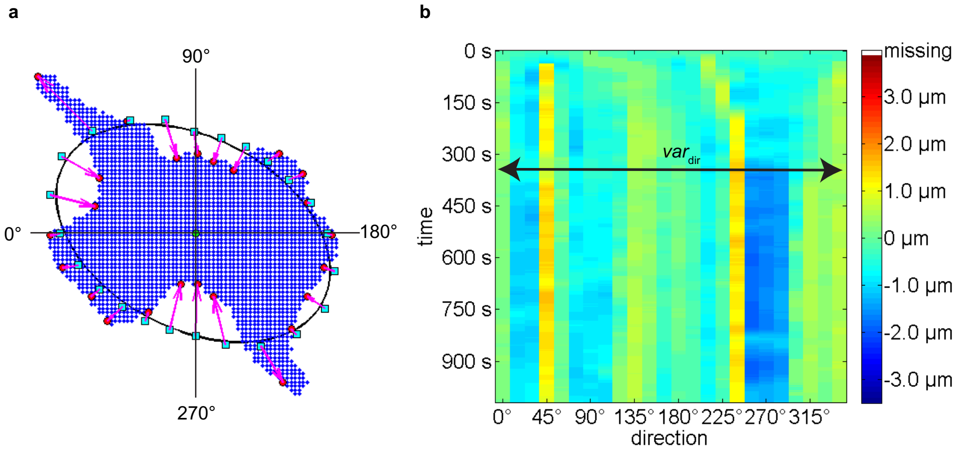

Figure S2: Analysis procedure of angular spreading dynamics. (a) MATLAB result for a typical cell (plot for one specific time point) that has responded to the structured substrate by avoiding the holes at its periphery. Invaginations and protrusions are shown by the magenta vectors $\vec{v}_{\text{cell-ellipse}}$ and the angles of 0°, 90°, 180° and 270° are indicated. (b) Color-coded plot of moving-average-treated signed lengths of vectors between cell and ellipse $d(n,t)$ with n being the angle denoted here on the $x-$axis and t the time-point in spreading denoted on the $y-$axis. The values for $d(n,t)$ are plotted ranging from dark blue for the largest negative values (invaginations, cell lies inside ellipse) and dark red for the largest positive values (protrusions, cell lies outside of ellipse). From the values of $d(n,t)$ the variance $var_{\text{dir}}(t)$ was calculated for each time-point.

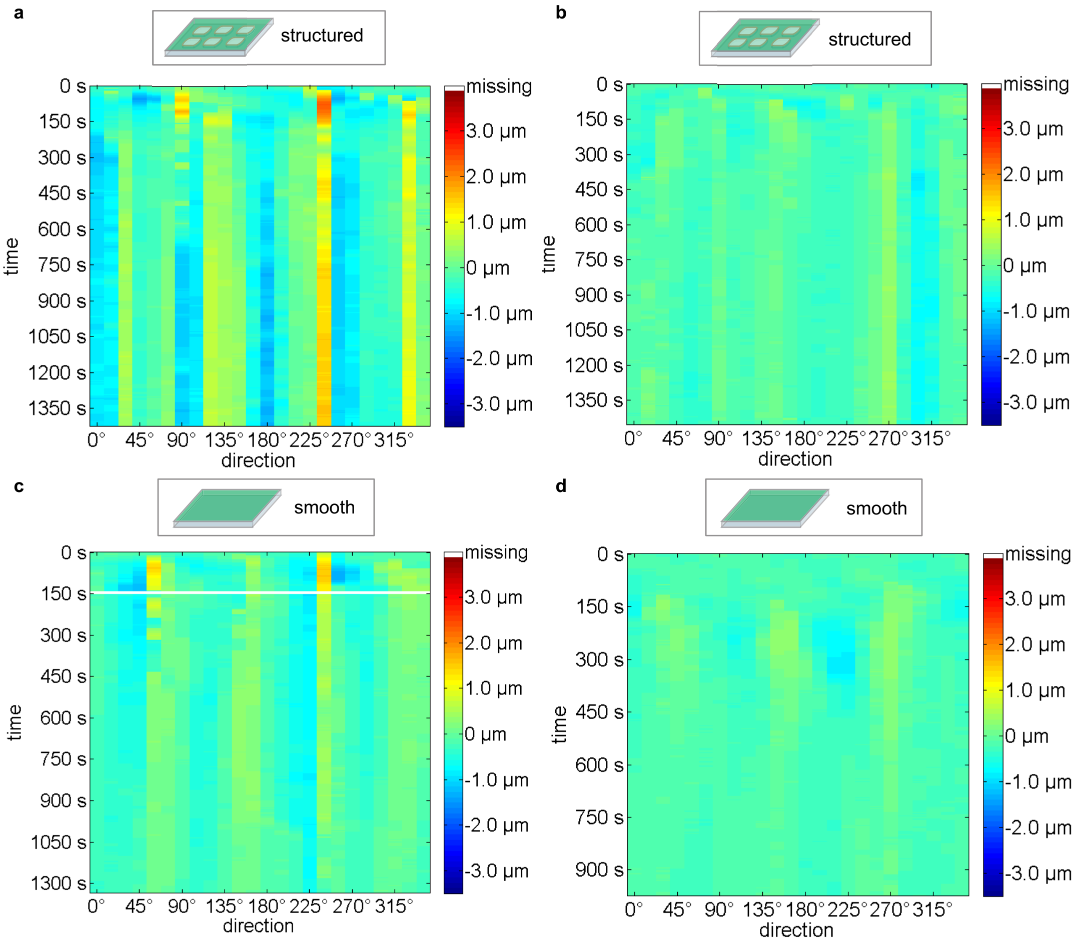

Figure S3: Color-coded plots of the moving average of the signed lengths of vectors between cell outline and ellipse $d(n, t)$ for different types of cells on structured ((a) and (b)) and smooth substrates ((c) and (d)). (a) The plot shows alternating protrusions and invaginations that become more pronounced with time. The alternating values indicate that the cell morphology is altered by the structured substrate. (b) Albeit also showing a cell on a structured substrate, the values fluctuate around zero with only one invagination at around 315°. This behavior indicates that the cell morphology is similar to that on smooth substrates. (c) This plot for a cell on a smooth substrate shows alternating protrusions and invaginations that even out over time indicating non-uniform spreading in the beginning followed by development of a smoother outline. (d) Similarly to (b) the values fluctuate around zero and have no larger order. Additionally, the differences disappear over time indicating an even cell outline as would be expected on a smooth substrate.

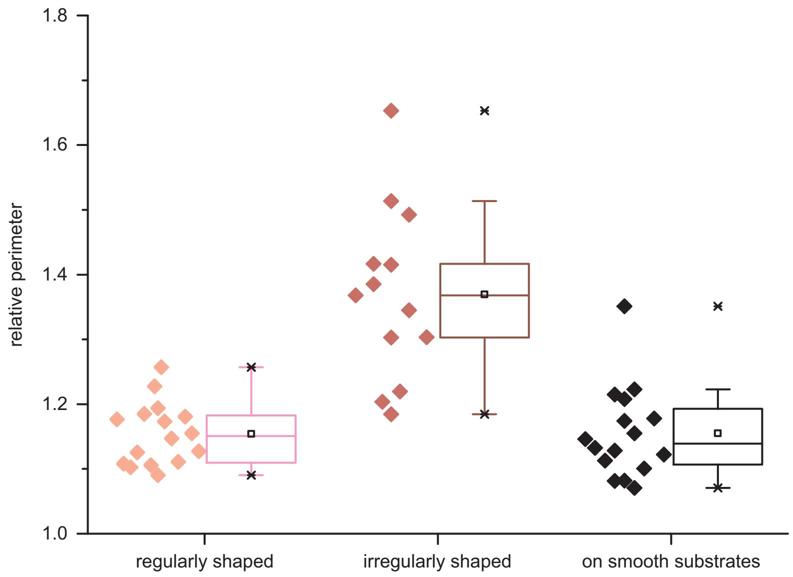

Figure S4: The relative perimeters ($perimeter_{cell}/perimeter_{ellipse}$) in the last analyzed time-point of the time-lapse series plotted for the different groups of cells (light orange: regularly shaped cells, pink: irregularly shaped cells, black: cells on smooth substrates). The relative perimeters for regularly shaped cells and cells on smooth substrates are quite similar while the relative perimeters for irregularly shaped cells are in general higher.

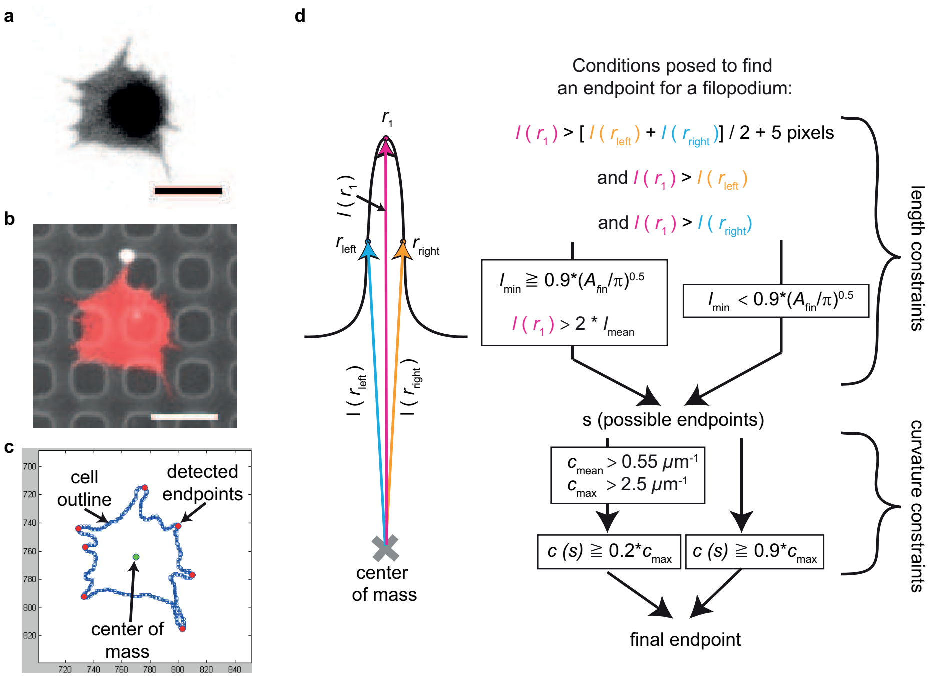

Figure S5: Filopodia detection in spreading platelets. (a) Inverted gray scale fluorescence micrograph (stained for plasma membrane, single frame of a movie) of a platelet spreading on a structured substrate and showing filopodia. (b) Overlay of fluorescence micrographs of membrane staining (red) and of the fibrinogen coating of the underlying substrate (gray). (c) Detected endpoints of filopodia depicted in red, the cell outline is shown in blue and the center of mass in green. (d) The cell outline (black), the center of mass of the cell (gray cross), the endpoint of a filopodium r_1, the points r_{left} and r_{right}, which lie 11 points left and right along the outline of point r_1 and the respective lengths from the center of mass to the above specified points $l(r_1)$ (pink), $l(r_{\text{left}})$ (blue) and $l(r_{\text{right}})$ (orange) are depicted. The lengths and curvature constraints that were posed to find an endpoint of a filopodium are sketched with l_{min} being the minimal length from center of mass to cell outline, A_{fin} being the area in the final image of spreading, l_{mean} being the mean length from center of mass to outline, c_{mean} being the mean curvature of all points of the outline and c_{max} being the maximum curvature of all points of the outline. Scale bars indicate 5 μm.

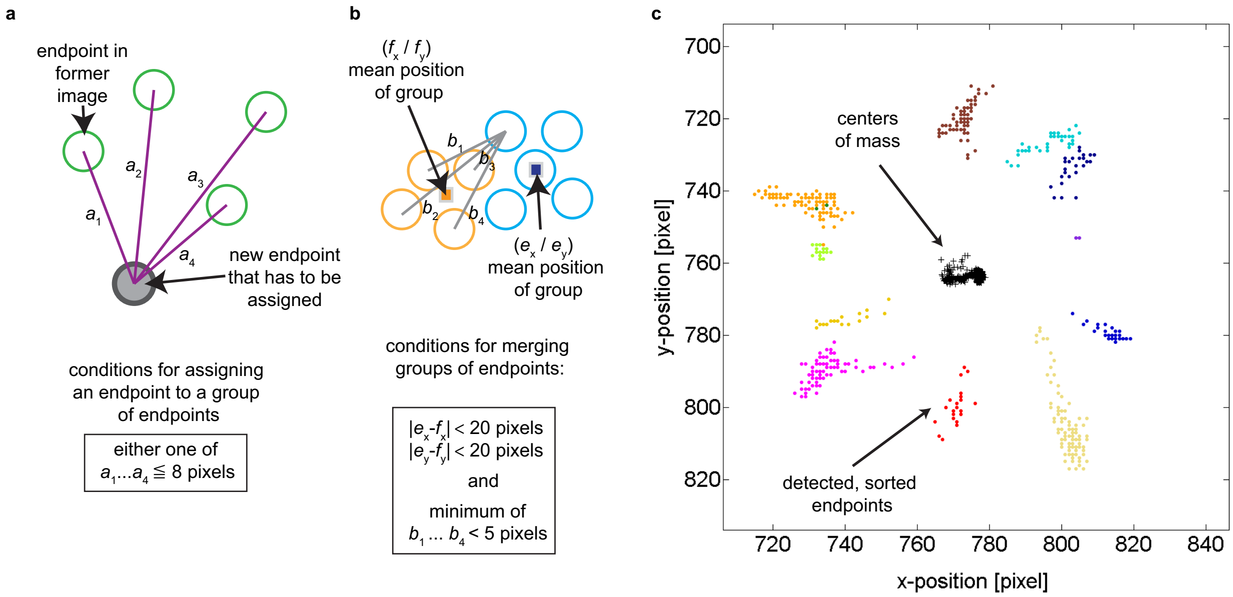

Figure S6: (a) Sketch of conditions under which an endpoint (gray) was assigned to a group of endpoints (green) from the former images. The distances between the points in the former images and the new point that has to be assigned are depicted in purple (a_1-a_4). If one of these distances is equal to or smaller than 8 pixels, the new endpoint is assigned to the group. (b) Here, two different groups of endpoints are depicted in orange and blue. The centers of mass of these groups are marked by a yellow and blue square, respectively. The distances between the points in the two groups are sketched here for one point of the blue group (b_1-b_4, gray lines). If the centers of mass are situated in close vicinity to each other and the smallest distance between the points of the two groups is smaller than 5 pixels, the groups are merged if the merging does not result in two endpoints being assigned simultaneously to a filopodium for one time point. (c) Endpoints of filopodia of the platelet depicted in figure S5 over the whole spreading process are shown in a color-coded way in order to show endpoints belonging to one filopod. The centers of mass for the individual frames are shown as black crosses.
